# EjWRKY6 Is Involved in the ABA-Induced Carotenoid Biosynthesis in Loquat Fruit during Ripening

**DOI:** 10.3390/foods13172829

**Published:** 2024-09-06

**Authors:** Yan Yu, Zeyang Bao, Qihang Zhou, Wei Wu, Wei Chen, Zhenfeng Yang, Li Wang, Xuewen Li, Shifeng Cao, Liyu Shi

**Affiliations:** 1 Zhejiang Key Laboratory of Intelligent Food Logistic and Processing, College of Biological and Environmental Sciences, Zhejiang Wanli University, Ningbo 315100, China; 17815963017@163.com (Y.Y.); baozy2022@163.com (Z.B.); zqh9952774810086@163.com (Q.Z.); wuwei@zwu.edu.cn (W.W.); vivianchanyee@zwu.edu.cn (W.C.); yangzf@zwu.edu.cn (Z.Y.); 2 School of Food Science and Pharmacy, Xinjiang Agricultural University, Urumqi 830052, China; xjndsp@sina.com; 3 College of Tea and Food Science and Technology, Anhui Agricultural University, Hefei 230036, China; lwang@ahau.edu.cn

**Keywords:** abscisic acid, loquat, carotenoids, EjWRKY6, transcriptional regulation

## Abstract

The yellow-fleshed loquat is abundant in carotenoids, which determine the fruit’s color, provide vitamin A, and offer anti-inflammatory and anti-cancer health benefits. In this research, the impact of abscisic acid (ABA), a plant hormone, on carotenoid metabolism and flesh pigmentation in ripening loquat fruits was determined. Results revealed that ABA treatment enhanced the overall content of carotenoids in loquat fruit, including major components like *β*-cryptoxanthin, lutein, and *β*-carotene, linked to the upregulation of most genes in the carotenoid biosynthesis pathway. Furthermore, a transcription factor, *EjWRKY6*, whose expression was induced by ABA, was identified and was thought to play a role in ABA-induced carotenoid acceleration. Transient overexpression of *EjWRKY6* in *Nicotiana benthamiana* and stable genetic transformation in *Nicotiana tabacum* with *EjWRKY6* indicated that both carotenoid production and genes related to carotenoid biosynthesis could be upregulated in transgenic plants. A dual-luciferase assay proposed a probable transcriptional control between EjWRKY6 and promoters of genes associated with carotenoid production. To sum up, pre-harvest ABA application could lead to carotenoid biosynthesis in loquat fruit through the EjWRKY6-induced carotenoid biosynthesis pathway.

## 1. Introduction

The loquat (*Eriobotrya japonica* Lindl.) is a subtropical evergreen tree whose fruit can be consumed fresh or processed [1]. Mature loquat fruit, belonging to the Rosaceae family, is characterized by carotenoids as its principal pigments [2,3]. The color of the fruit distinguishes red and white-fleshed loquat varieties, with red-fleshed varieties exhibiting a reddish hue due to their high carotenoid content, while white-fleshed loquats have a lower carotenoid content [4,5]. The ripening of loquat fruit is marked by carotenoid biosynthesis-induced color transformation and changes in fruit firmness [6,7]. Carotenoids exhibit diverse functions across animals, plants, and microorganisms. In plants, carotenoids serve as light collectors for photosynthesis, protecting them from excessive light stress, and adding vibrant colors to flowers and fruits, thereby attracting animals for pollination and facilitating seed dispersal [8]. As for human and animal health, carotenoids offer essential nutrients, with beta-carotene being a primary source of vitamin A, synthesized naturally within our bodies. Vitamin A is indispensable for vision, bolstering immunity against infectious diseases, preserving epithelial cell integrity, facilitating bone remodeling, and supporting reproduction. Its antioxidant prowess contributes to immune regulation and guards against cancer, cardiovascular diseases, and ocular ailments, as demonstrated in various studies [9,10,11]. Furthermore, dietary carotenoid intake diminishes the likelihood of developing numerous chronic conditions. Additionally, carotenoids play a pivotal role in mediating plants’ responses to environmental cues, demonstrating their multifaceted importance [12].

The carotenoid biosynthesis pathway in higher plants begins with the 2-methyl-D-erythritol-4-phosphate (MEP) pathway as outlined by Eisenreich et al. [13]. The pathway produces geranylgeranyl diphosphate (GGPP), which forms the initial carotenoid, octahydro lycopene, through the catalysis of the enzyme petrosyllabin synthase (PSY) [14]. Red lycopene is produced through a series of desaturation and isomerization reactions of octahydrolycopene facilitated by enzymes such as octahydrolycopene desaturase (PDS), *ζ*-carotene desaturase (ZDS), *ζ*-carotene iso (Z-ISO), and carotenoid iso (CRTISO). Lycopene then enters two derivatization pathways to generate different derivatives, namely the *α*-branch and *β*-branch pathways. In the *α*-branching pathway, lycopene is cyclized to alpha-carotene through either beta-cyclization (LCYB) or *ɛ*-cyclization (LCYE), followed by hydroxylation to lutein by two heme-free carotene hydroxylases (BCH1 and BCH2) and two hemoglobin hydroxylases (CYP97A and CYP97C). Within the *β*-branch, lycopene is cyclized by LCYB into *β*-carotene, which is subsequently converted to zeaxanthin via hydroxylation by BCH1 and BCH2. Zeaxanthin forms the xanthophyll cycle in the presence of zeaxanthin epoxidase (ZEP), which protects plants from light damage [15,16]. The breakdown is regulated by carotenoid cleaving dioxygenases (CCDs) and 9-cis-epoxycarotenoid dioxygenases (NCEDs), contributing to maintaining the dynamic equilibrium of carotenoids and the production of abscisic acid (ABA). Su et al. [17] found that carotenoid accumulation in ‘Jiefangzhong’ loquat was closely related to the strong expression of *PSY*, *ZDS*, and *ZEP*. Differences in carotenoid accumulation among red-fleshed loquat (‘Luoyangqing’) and white-fleshed loquat (‘Baisha’) were primarily related to differences in the expression of *PSY1* and *BCH* [3]. Additionally, high expression of the *LCYB* gene allowed red-fleshed loquat varieties to obtain more carotenoids [5].

Recently, many transcription factors have been implicated in the regulation of carotenoids. For example, MdMADS6 can activate *MdCCD1* by binding with CArG elements, promoting carotenoid accumulation in apple fruit [18]. In citrus, CsMADS6 promoted carotenoid synthesis by directly binding with the promoters of *PDS* and *CCD1* genes associated with high carotenoid synthesis [19]. During papaya ripening, CpNAC1 regulated carotenoid biosynthesis by activating *CpPDS2/4* [20]. A recent study reported that MiMYB10 could control mango peel color by activating the expressions of carotenoid biosynthesis genes *PSY*, *ZISO*, *CRTISO*, and *LCYE*, which promote carotenoid accumulation [21]. WRKY transcription factors are a class of transcription regulatory factors that are unique and functionally diverse in higher plants. They regulate gene expression by specifically binding to the cis acting element W-box of target gene promoters and participate in various biotic and abiotic stress responses [22]. In addition, WRKY transcription factors also play an important role in the accumulation of plant pigments. Duan et al. [23] revealed that CmWRKY49, had a direct connection to the *CmPSY1* promoter, thereby increasing the β-carotene content in orange-coloured Oriental melons. However, limited information is available about the transcriptional regulation of carotenoid biosynthesis in loquat fruit.

Abscisic acid (ABA) was initially identified in young cotton fruits and dormant bubinga buds [24,25,26]. Ranked among the top five natural plant growth regulators, ABA exhibits potent functions such as promoting abscission, inducing stomatal closure, and enhancing plant resistance [27]. The capacity of ABA to regulate growth and color in fruit further emphasizes its major role in fruit ripening and senescence [28]. Exogenous ABA treatment has been demonstrated to augment the activities of anthocyanin biosynthetic enzymes such as PAL and TAL, leading to elevated anthocyanin content and enhanced color formation in strawberries [29]. The expression of carotenoid biosynthesis genes *PSY1*, *PDS*, and *ZDS* was significantly increased by exogenous ABA treatment in tomatoes, thus facilitating the acceleration of carotenoid production and encouraging early color change [30]. Furthermore, ABA treatment induced an upregulation of the plant-specific regulatory gene *SISGR1* in tomatoes, a gene involved in chlorophyll degradation. SISGR1 promoted chlorophyll degradation by interacting with SIPPH and SLHCa2, thus contributing to ABA-induced chlorophyll degradation and color development in tomatoes [31]. However, the precise role of ABA in modulating carotenoid production in loquat fruit remains unclear.

Therefore, this study aims to explore the impact and mechanisms of exogenous ABA on carotenoid metabolism during the ripening of loquat fruit. Through transcriptome sequencing, we identified a WRKY family transcription factor, EjWRKY6, which showed significantly increased expression in response to ABA (unpublished results). The primary objective of this research is to further elucidate the transcriptional regulatory mechanism by which EjWRKY6 influences carotenoid accumulation in loquat.

## 2. Materials and Methods

### 2.1. Plant Materials and Treatments

Three loquat (*Eriobotrya japonica* cv. Dahongpao) trees were selected from an orchard in Xiangshan, Zhejiang Province. Their fruits were treated 92–98 days after full bloom (DAFB). The fruits on each tree were divided into two groups: one group was sprayed with 66.08 mg L^−1^ ABA (dissolved in distilled water), while the other group was sprayed with distilled water as a control. After spraying, the fruits were air-dried naturally and then individually covered with double-layered, fruit-specific bags. The fruits were initially picked as 0-day samples prior to the spraying treatment. Subsequently, they were harvested at 3, 4, 5, and 6 days after each spray. At each time point, 20 fruits were individually harvested from both the ABA treatment group and the control group of each tree, and thereafter, the fruits from the ABA treatment group and the control group across the 3 trees were merged separately. The color difference and firmness of the fruits were measured, and the fruit pulp was frozen using liquid nitrogen and stored at −80 °C for further analysis.

### 2.2. Fruit Firmness and Color Measurement

Firmness was assessed using a texture analyzer, TMS-Touch (Federal Trade Commission, Wshington, DC, USA), equipped with a probe diameter of 7.5 mm and a test rate of 1 mm s^−1^. Measurements were taken at equatorial positions on both sides of the fruit. The results were reported as the average force in Newtons (N) from five fruits in each biological replicate. The peel and flesh color were assessed by taking measurements at three uniformly distributed points along the equator of the fruit with a colorimeter (Konica Minolta, CM-26D, Tokyo, Japan). Five fruit color differences were measured in each biological replicate. The results were presented as the mean of a*/b* ratios to represent the color indices, following the method by Alos et al. [32].

### 2.3. Extracting and Quantifying Carotenoid

Total carotenoids of loquat pulp were determined by spectrophotometry [33], and carotenoid fractions were measured using HPLC [33]. The process for extracting total carotenoids, including both esterified and free carotenoids, was slightly modified following the method described by Tuan et al. [34]. Approximately 1 g of the powder sample ground with liquid nitrogen was soaked for 10 h with 5 mL of absolute ethyl alcohol dissolved with 0.1% ascorbic acid and pre-cooled at −20 °C for 30 min. After extraction, 300 μL of 80% potassium hydroxide solution was added, and saponification was conducted in the darkness at 95 °C for 45 min. The saponification reaction was terminated by adding 5 mL of pre-cooled water. Following this, purification of the saponification blend was achieved by incorporating 5 mL of petroleum ether, which was then spun at 4 °C, 6000× *g*, for 10 min. The remaining petroleum ether was extracted 4–5 times until the carotenoid derivatives in the mixture were completely extracted, and the combined extracts were collected. After drying under N_2_ at 35 °C, the final solution was dissolved by adding methanol/dichloromethane (1:1), and this solution was utilized for the HPLC analysis of the carotenoid fractions and the spectrophotometric measurement of the total carotenoid content.

The carotenoid fractions were determined using a Waters E2695 model machine (Milford, MA, USA) equipped with a C30 chromatographic column (3 μm, 250 × 4.6 mm; YMC Corporation, Kyoto, Japan) at a temperature of 40 °C and a flow rate of 10 μL s^−1^. The mobile phases consisted of methyl tert-butyl ether (elution fluid A), methanol (elution fluid B), and H_2_O (elution fluid C), following a stepwise elution procedure over time: 10% A, 86% B, and 4% C at 0 min; 15% A, 71% B, and 4% C at 10 min; 90% A, 6% B, and 4% C at 50 min; 10% A, 86% B, and 4% C at 53 min. At the detection wavelength of 450 nm, lutein, β-cryptoxanthin, and β-carotene were separated with distinct retention times (17.40 min for lutein, 25.85 min for β-cryptoxanthin, and 33.52 min for β-carotene), and subsequently, they were individually quantified using standard curves plotted based on their respective standard components.

### 2.4. Gene Expression Analysis by Real-Time Quantitative PCR (qPCR)

The gene expression analysis was carried out in the pulp of loquat and in the leaves of tobacco. Total RNA extraction and cDNA synthesis were performed according to Zhang et al. [35]. Gene expression analysis was carried out using the Bio-Rad CFX96 real-time system (Bio-Rad, Hercules, CA, USA) employing the ChamQ Universal SYBR qPCR Master Mix reagent (Vazyme, Nanjing, China), which incorporates Champagne Taq DNA Polymerase (Vazyme, Nanjing, Jiangsu, China). The specific primers used for qPCR are listed in Supplementary Table S1. They were designed using beacon designer 7.9 software with the parameters of melting temperature (Tm) at 55.0 ± 5.0 °C, primer length ranging from 19 to 22 bp, and amplification product length between 75 and 200 bp. The gene expression was calculated using the 2^−ΔCT^ method, with *EjACT* as the reference gene for loquat and *NbEF1α* as the reference gene for tobacco. Four biological replicates were performed for each sample.

### 2.5. Transient Overexpression in Tobacco

The open reading frame (ORF) sequence of *EjWRKY6* was inserted into the pGreen II 0029 62-SK vector [36], with the primers listed in Supplementary Table S2. The target gene and empty vector were separately transformed into *Agrobacterium rhizogenes* GV3101 (Shanghai Vidi Biotechnology Co., Ltd., Shanghai, China) containing pSoup-P19 and then infiltrated into both sides of tobacco leaves at a re-suspended concentration of OD_600_ = 1. After 72 h of cultivation, the infected areas of tobacco leaves were sampled to determine the relative expression levels of *EjWRKY6* and carotenoid synthesis genes. The total carotenoid content in tobacco leaves was determined according to the method of Liu et al. [33].

### 2.6. Genetic Transformation of Tobacco

The *EjWRKY6* ORF sequence was inserted into the PRI101 vector and transferred into *Agrobacterium tumefaciens* strain EHA105. The primers used were listed in Supplemental Table S2. After cultivating the bacteria to an OD600 of 1, the solution was used to soak flue-cured tobacco (*Nicotiana tabacum*) leaf segments (0.5 cm × 0.5 cm) for 15 min [37]. The leaves were then cultured for two days on MS medium supplemented with 0.2 mg L^−1^ NAA and 2.0 mg L^−1^ 6-BA. Subsequently, the infected leaves were transferred to a regeneration MS medium containing 0.2 mg L^−1^ NAA, 2.0 mg L^−1^ 6-BA, 50 mg L^−1^ kanamycin, and 300 mg L^−1^ cefotaxime. After one month of growing adventitious buds, the carotenoid content and related gene expression levels in the transgenic tobacco leaves were determined.

### 2.7. Dual-Luciferase Assays

The *EjWRKY6* ORF sequence was inserted into the pGreen II 0029 62-SK vector, and the pGreen II 0800-LUC vector was fused with the promoter of the carotenoid biosynthetic gene [36]. Primers as shown in Supplementary Table S2 were used. According to the method described by Li et al. [38], the recombinant reporter gene and the effector gene were separately transformed into *Agrobacterium tumefaciens* GV3101 containing pSoup-P19 (Shanghai Weidi Biotechnology, Shanghai, China). The suspension of the infiltrated bacteria was then injected into *Nicotiana benthamiana* leaves, and after 72 h of cultivation, the Dual-Luciferase Assay Kit (Vazyme) was used to analyze the ratio of firefly luciferase (LUC) to Renilla luciferase (REN) activity.

### 2.8. Statistics Analysis

GraphPad Prism 9 software was employed to analyze the experimental data. Multiple *t*-tests were used for differences between the control and treatment groups (* *p* < 0.05, ** *p* < 0.01, and *** *p* < 0.001). Figures were made by GraphPad Prism 9 and Adobe Photoshop CS6.

## 3. Results

### 3.1. Effect of ABA Treatment on Color and Hardness of Loquat Fruits

**Figure 1:**
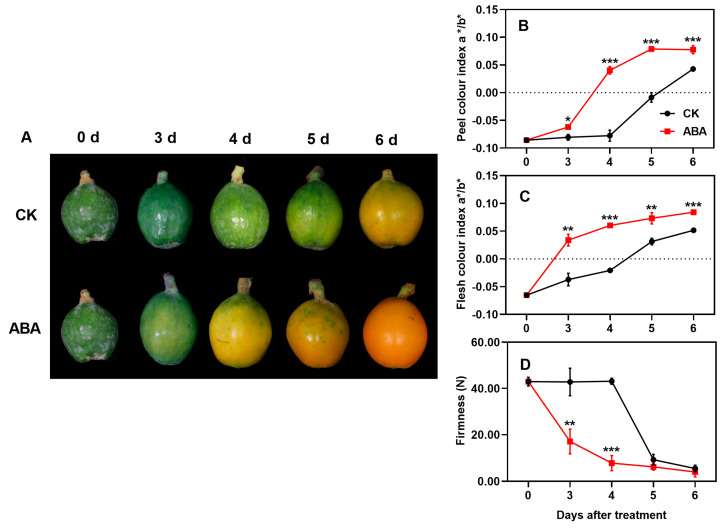
Effect of ABA treatment on loquat appearance (**A**), peel color difference index (**B**), flesh color difference index (**C**), and firmness (**D**). Asterisks indicate significant differences between CK and ABA treatment groups (* *p* < 0.05, ** *p* < 0.01, and *** *p* < 0.001). The Y-bars shown on the data points represent the standard deviation (SD).

As shown in Figure 1A, loquat fruits transitioned from green to yellow as they matured, with the yellow color gradually intensifying. Notably, ABA-treated loquat fruits began their color transformation and turned yellow by the third day, whereas the control fruits started this process on the fifth day. By the sixth day of ABA treatment, the treated fruits were completely yellow, with a significantly higher intensity of yellow compared to the controls. The color indices a*/b* for both the peel and flesh of loquat fruits exhibited an increasing trend during ripening, with ABA-treated fruits consistently showing higher values than the controls (Figure 1B,C). Concurrently, fruit firmness gradually declined during ripening, with ABA treatment enhancing this process (Figure 1D).

### 3.2. Effect of ABA Treatment on Carotenoid Content and Composition of Loquat Fruits 

**Figure 2:**
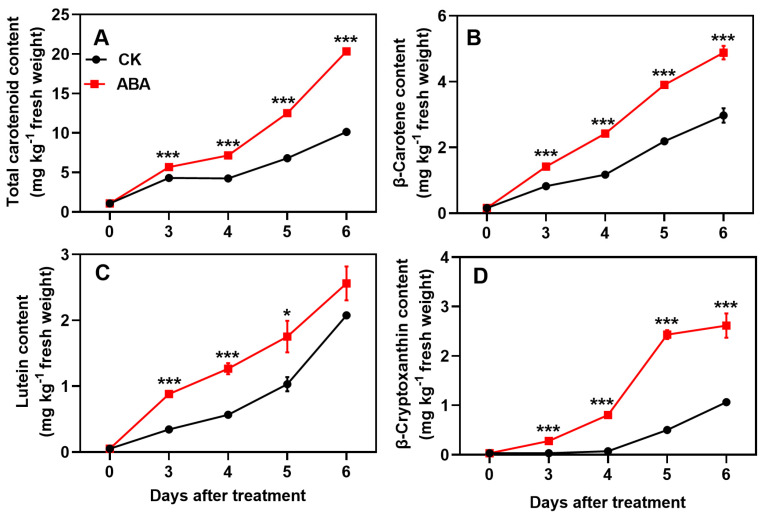
Effect of ABA treatment on total carotenoid (**A**), *β*-carotene (**B**), lutein (**C**), and *β*-cryptoxanthin (**D**) content of loquat pulp. Asterisks indicate significant differences between CK and ABA treatment groups (* *p* < 0.05 and *** *p* < 0.001). The Y-bars shown on the data points represent SD.

The total carotenoid content showed a gradual increase during the ripening of loquat fruits, with ABA application further enhancing this rise (Figure 2A). HPLC analysis revealed that *β*-carotene was the primary carotenoid present in loquat fruit, along with lutein and *β*-cryptoxanthin. The levels of these main carotenoids increased as the fruits ripened, with ABA treatment significantly boosting their accumulation (Figure 2B–D).

### 3.3. Effects of ABA Treatment on the Expression of Carotenoid Biosynthesis Genes in Loquat Fruits

**Figure 3:**
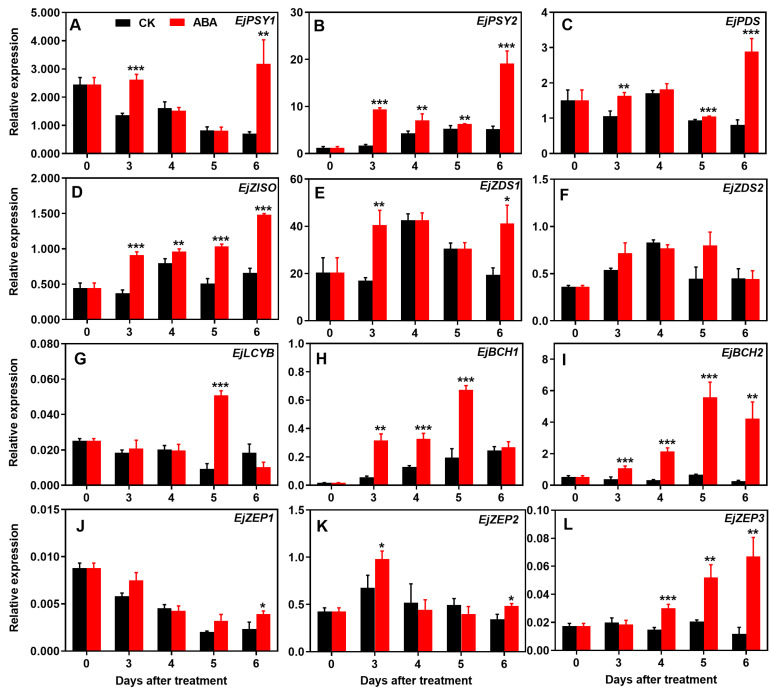
Effect of ABA treatment on the expression of carotenoid biosynthesis-related genes in loquat. The expression levels of *EjPSY1* (**A**), *EjPSY2* (**B**), *EjPDS* (**C**), *EjZISO* (**D**), *EjZDS1* (**E**), *EjZDS2* (**F**), *EjLCYB* (**G**), *EjBCH1* (**H**), *EjBCH2* (**I**), *EjZEP1* (**J**), *EjZEP2* (**K**), and *EjZEP3* (**L**) were analyzed. Asterisks indicate significant differences between CK and ABA treatment groups (* *p* < 0.05, ** *p* < 0.01, and *** *p* < 0.001). The Y-bars shown on the data points represent SD.

A decline of the transcript abundance of *EjPSY1* was observed in the non-treated loquat fruit during ripening; however, its expression level was significantly upregulated on days 3 and 6 after ABA treatment (Figure 3A). The expression of *EjPSY2* showed an increasing trend during maturation, and ABA treatment significantly increased its transcript level during ripening (Figure 3B). The expressions of *EjPDS*, *EjZISO*, and *EjZDS1/2* in the non-treated loquats first increased and then declined. The expression of *EjPDS* and *EjZISO* was notably increased by ABA treatment during ripening, with the exception of *EjPDS* on day 4 (Figure 3C,D). On the other hand, the expression of *EjZDS1* was upregulated by ABA treatment at 3 and 6 days (Figure 3E). However, the transcript of *EjZDS2* did not show significant differences under ABA treatment (Figure 3F). A higher *EjLCYB* transcript abundance was only observed on day 5 in the ABA-treated loquats (Figure 3G); however, the treatment significantly increased the transcript level of *EjBCH1/2* during ripening, with an exception on day 6 for *EjBCH1* (Figure 3H,I). The expression of three *EjZEPs* in the non-treated loquats experienced different changes during ripening. ABA upregulated *EjZEP1* only at the end of ripening but *EjZEP2* on days 3 and 6 (Figure 3J,K). However, the transcript levels of *EjZEP3* were obviously higher after 4 days with ABA treatment than the control (Figure 3L).

### 3.4. Effects of ABA Treatment on EjWRKY6 Expression in Loquat Fruits

**Figure 4:**
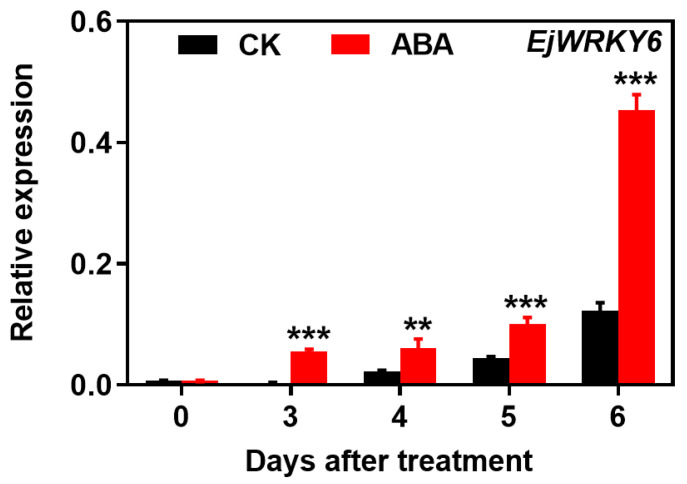
Effects of ABA treatment on the expression of *EjWRKY6* transcription factors in loquat. Asterisks indicate significant differences between CK and ABA treatment groups (** *p* < 0.01 and *** *p* < 0.001). The Y-bars shown on the data points represent SD.

Screened within the transcriptome sequencing database of ABA-treated loquat fruit (unpublished results), a WRKY transcription factor EjWRKY6, exhibiting high transcript levels, was selected. qPCR validation confirmed a gradual rise in the transcript levels of *EjWRKY6* during the ripening process in both untreated and treated loquats, with ABA treatment further inducing its expression (Figure 4).

### 3.5. Transient Overexpression of EjWRKY6 in Nicotiana benthamiana

**Figure 5:**
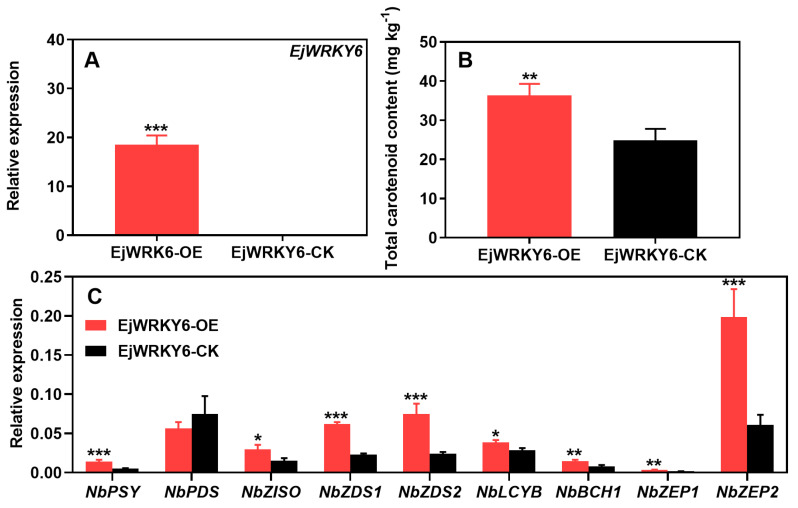
Effect of *EjWRKY6* treatment on carotenoid content and expression of biosynthesis-related genes in transiently overexpressed tobacco. The expression of *EjWRKY6* (**A**), tobacco carotenoid content (**B**), and the expression of *NbPSY*, *NbPDS*, *NbZISO*, *NbZDS1*, *NbZDS2*, *NbLCYB*, *NbBCH1*, *NbZEP1*, and *NbZEP2* (**C**) were analyzed. Asterisks indicate significant differences between CK and OE groups (* *p* < 0.05, ** *p* < 0.01, and *** *p* < 0.001). The Y-bars shown on the data points represent SD.

To explore the potentially relevant correlation between EjWRKY6 and carotenoid regulation, agrobacterium-mediated transient expression of *EjWRKY6* was conducted in *Nicotiana benthamiana* leaves (Figure 5A). The results revealed that the transient expression of *EjWRKY6* (OE) resulted in higher total carotenoid content compared with empty vector plants (CK) (Figure 5B). The expression of the majority of carotenoid biosynthetic genes was upregulated by the overabundance of *EjWRKY6*, thus leading to an increase in the overall carotenoid levels (Figure 5C). However, the overexpression had no influence on *NbPDS* expression (Figure 5C).

### 3.6. Stable Overexpression of EjWRKY6 in Nicotiana tabacum

**Figure 6:**
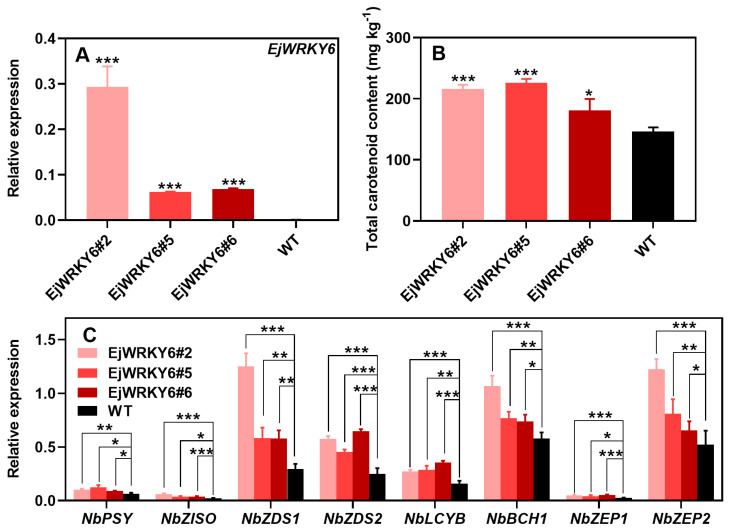
Effect of EjWRKY6 on carotenoid content and expression of biosynthesis-related genes in stable genetic transgenics of tobacco. The expression of *EjWRKY6* (**A**), tobacco carotenoid content (**B**), and the expression of *NbPSY*, *NbZISO*, *NbZDS1*, *NbZDS2*, *NbLCYB*, *NbBCH1*, *NbZEP1*, and *NbZEP2* (**C**) were analyzed. Asterisks indicate significant differences between CK and OE groups (* *p* < 0.05, ** *p* < 0.01, and *** *p* < 0.001). The Y-bars shown on the data points represent SD.

Due to the challenge of transformation in loquat fruit, the expression of *EjWRKY6* was introduced in *Nicotiana tabacum* to evaluate the in vitro effects of *EjWRKY6*. Of the eight T2 transgenic plants, those numbered 2, 5, and 6 had high expression levels of *EjWRKY6* and were therefore selected for in-depth study (Figure 6A). During the growth process, no significant phenotypic differences were observed between the wild-type (WT) and the three transgenic plants. Total carotenoids in these transgenic tobacco plants were found to be greater than in the WT plants (Figure 6B). Importantly, the expression levels of most carotenoid biosynthetic genes in the transgenic tobacco plants, such as *NbPSY*, *NbZISO*, *NbZDS1*, *NbZDS2*, *NbLCYB*, *NbBCH1*, *NbZEP1*, and *NbZEP2*, were significantly upregulated when compared to the WT plants (Figure 6C).

### 3.7. Transcriptional Regulation on EjWRKY6

**Figure 7:**
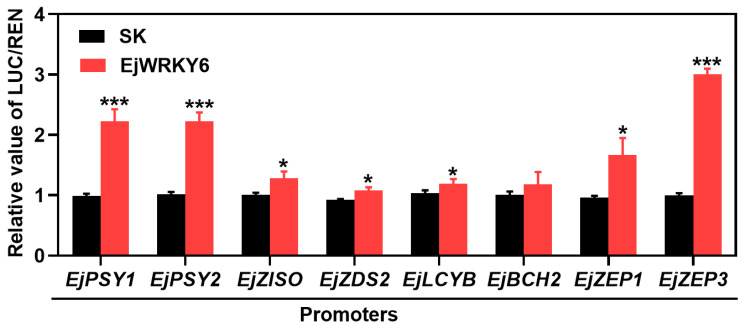
Transcriptional regulation of EjWRKY6 analyzed by a dual-luciferase assay. The regulation effects of *EjPSY1*, *EjPSY2*, *EjZISO*, *EjZDS2*, *EjLCYB*, *EjBCH2*, *EjZEP1*, and *EjZEP3* by EjWRKY6 were analyzed. Asterisks indicate significant differences between the empty vector group (SK) and the transient expression group of EjWRKY6 (* *p* < 0.05 and *** *p* < 0.001). The Y-bars shown on the data points represent SD.

A dual-luciferase assay was employed to further investigate the role of EjWRKY6 in regulating the promoters of eight carotenoid biosynthetic genes, which were successfully cloned. The results showed that, with the exception of *EjBCH2*, EjWRKY6 could activate the promoter activity of all the other genes examined, thereby enhancing their transcription (Figure 7).

## 4. Discussion

Carotenoids are recognized as the primary pigments in most fruit crops, with their synthesis and breakdown pathways influenced by hormones [16,39]. Ethylene, particularly during ripening, has been observed to increase carotenoid levels, including *β*-carotene and *β*-cryptoxanthin in loquat fruit [32]. Sun et al. [40] revealed that melatonin treatment significantly induced carotenoid content, including *α*, *β*-carotene, and lycopene in cherry tomato fruits. Conversely, indole-3-acetic acid treatment decelerated the accumulation of carotenoids, while its antagonist PCIB enhanced it [41]. Recent research by Ma et al. [42] demonstrated that applying ABA externally increased total carotenoid content, especially *β*-xanthophylls, in citrus fruit. Similarly, the present study indicated that pre-harvest treatment with ABA could increase carotenoid accumulation, particularly major fractions like lutein, *β*-cryptoxanthin, and *β*-carotene, in loquat fruit during ripening.

Exogenous hormones regulate the transcription of structural genes involved in carotenoid synthesis, thereby influencing carotenoid accumulation. Ethylene, for example, upregulated *LCYB* gene expression in durian pulp, leading to increased carotenoid content, including *β*-carotene and *α*-carotene. Conversely, 1-MCP (1-Methylcyclopropene) treatment decreases the expression of *ZDS*, *LCYB*, *CYCB*, and *BCH*, delaying the carotenoid accumulation [43]. Methyl jasmonate and brassinosteroid induced lycopene accumulation and overall carotenoid content in tomato fruits by upregulating specific transcripts in the carotenoid biosynthesis pathway [44]. In contrast, gibberellin treatment inhibited carotenoid synthesis in orange fruit by downregulating the transcription of *CitPSY*, *CitPDS*, *CitZDS*, and *CitLCYB2*, inducing fruit re-greening [45]. Our research here suggested that ABA treatment increased the transcription of most genes associated with carotenoid biosynthesis. The upregulation of genes like *EjPSY1/2*, *EjPDS*, *EjZISO*, and *EjZDS1/2* under ABA treatment contributed to enhanced *β*-carotene production. Additionally, in ABA-treated loquats, elevated gene expression of *EjLCYB* was responsible for higher lutein levels, while increased *β*-cryptoxanthin content was linked to ABA-induced upregulation of *EjBCH1/2*.

WRKYs are one of the most expansive and extensively researched families of transcription factors in plants [46]. They play pivotal roles in regulating various aspects of plant life, including seed development, germination, flowering, and responses to environmental stresses [47,48,49,50,51,52]. Recently, WRKY transcription factors have been revealed as major regulators for fruit pigmentation. In tomato fruits, eight SIER-WRKY transcription factors have been identified to promote lycopene accumulation by activating the *SIPSY1* and *SIPDS* promoters [53]. In pear, PbWRKY75 had the capability to stimulate anthocyanin production by activating *PbDFR* and *PbMYB10b* [54]. Additionally, the PyWRKY26 and PybHLH3 interaction targeted the *PyMYB114* promoter, controlling both the biosynthesis and transportation of anthocyanin in red pears, ultimately enhancing anthocyanin accumulation [55]. A transcription factor EjWRKY6 from the WRKY family was screened to reveal the regulatory process of carotenoid metabolism in loquat fruit. Both transient overexpression in *Nicotiana benthamiana* and stable transformation in *Nicotiana tabacum* with *EjWRKY6* demonstrated that EjWRKY6 could elevate the total carotenoid content by upregulating carotenoid-related biosynthetic genes, suggesting EjWRKY6 as an enhancer for carotenoid synthesis in loquat fruit during ripening.

WRKY transcription factors have been demonstrated to bind to the W-box site of target genes, thereby influencing plant responses [22]. For instance, DkWRKY7 directly interacted with the promoter of *DkPDC2* at the W-box site, regulating persimmon fruit deastringency [56]. Similarly, HpWRKY44 was bound to the promoter of *Cytp450-like1* at the W-box, playing a role in betalain biosynthesis in pitaya fruit [57]. In the present study, dual-luciferase assays showed that EjWRKY6 activated the promoters of all eight structural genes involved in carotenoid biosynthesis, suggesting a potential direct and indirect regulatory mechanism by EjWRKY6 on these gene promoters.

## 5. Conclusions

In conclusion, exogenous ABA treatment promoted the accumulation of total carotenoids and major carotenoid components, including *β*-carotene, *β*-cryptoxanthin, and lutein, in loquat pulp. ABA enhanced carotenoid accumulation by regulating the expression of genes related to carotenoid biosynthesis in loquat fruit. Moreover, our findings indicated the potential involvement of the candidate EjWRKY6 transcription factor in carotenoid accumulation. Both transient and transgenic overexpression of EjWRKY6 increased the carotenoid content in tobacco leaves and enhanced the expression of carotenoid synthesis genes. A dual-luciferase assay confirmed that EjWRKY6 can activate the promoter of carotenoid synthesis genes. These results indicate that EjWRKY6 acts as a positive regulator, mediating ABA-induced carotenoid accumulation in loquat fruit. However, the specific regulatory role of this transcription factor in carotenoid biosynthesis in loquat fruit needs further investigation. Such endeavors will significantly contribute to a deeper comprehension of the carotenoid accumulation mechanisms in loquat fruits, thereby bolstering the genetic enhancement and quality upgrading of related crops.

## Data Availability

The original contributions presented in the study are included in the article/Supplementary Material; further inquiries can be directed to the corresponding authors.

## Supplementary Materials

The following supporting information can be downloaded at https://www.mdpi.com/article/10.3390/foods13172829/s1: Table S1: Primer sequences used for RT-qPCR analysis; Table S2. Primer sequences used for vector construction.
