# Supplementary material for: EjWRKY6 Is Involved in the ABA-Induced Carotenoid Biosynthesis in Loquat Fruit during Ripening
Source: Foods. 2024 Sep 6;13(17):2829. doi: 10.3390/foods13172829 (PMC11395680; doi:10.3390/foods13172829)
Supplement: Supplementary file 1 [file foods-13-02829-s001.zip › supplementary Table S1.pdf]

Table S1. Primer sequences used for RT-qPCR analysis

Gene name	Forward Primer	Reverse Primer
EjPSY1	GAAGGATCAAATTAAGAGGG	TTGGCGATATAGGAGTAG
EjPSY2	GACTGACTGATCTCTTTG	TCTGGTATCTTGACTTCC
EjPDS	CCAAGGTCAGTTTACAAG	CAGCAAGCAATTCATAATC
EjZISO	GGGAAACTGGGATTATGA	CCTCCCCGTATCTTATAG
EjZDS1	GCTTCTTGGGCTCTTTTAC	AGGAGGAGGACCTCTTTG
EjZDS2	ACACCACATTTTGATTTGA	GAGCTGAGTCTTGTCTAA
EjLCYB	GAGAGTTGTTGGAATCGG	GGTACTGAACTATTGCATTTG
EjBCH1	CATGGACAAACATATACGC	CTCCTTAATTACGGTTTCTTC
EjBCH2	ATCGGAGAGGTTCACTTA	CCCAGCATTTCAGACATA
EjZEP1	AAGGCTGAATTGGATTTG	GCTTCTTTGTGCATACTC
EjZEP2	CGAACATGGTTTGAAGAC	GCCCACAAGGTATAAGATA
EjZEP3	ACGCCATTGTTATTGCTC	CTCTCTTTCACGTACTTGTC
EjACT	CTGTGTTTCCTAGTATTGTTG	CTGTGCTTCATCACCTAC
EjWRKY6	CTATCACCTCCATCATTG	ATGTTCTTGTTGTTGTTG
NbPSY	TATTCAGCCATTCAGAGA	CGTAATAACAATATAGGTATAGC
NbPDS	ATCTGATAATCTGCTCTTC	CCAATTCCAACATAGACT
NbZISO	TTCCTTCTTCTTCCTATAC	AATCCTCATAATTCCAGTT
NbZDS1	TGATGGAAGTATGTATGTT	CATTGTATCGTAGTTGTAC
NbZDS2	TGATGGAAGTATGTATGTT	CATTGTATCGTAGTTGTAC
NbLCYB	GGACGAGTATTGTGTAAT	GCTACCATATAACCAGTT
NbBCH1	CAGAGGAGATTGAATTGA	CATAATAGCCAAGGAAGT
NbZEP1	AGAACTCAATTCAGAACAT	CTCCAATCAACACTACTT
NbZEP2	ATTAGAGGAGAAGGACAA	CAATACCAGTTACCAGAA
NbEF1α	AGAGGCCCTCAGACAAAC	TAGGTCCAAAGGTCACAA
