# Supplementary material for: EjWRKY6 Is Involved in the ABA-Induced Carotenoid Biosynthesis in Loquat Fruit during Ripening
Source: Foods. 2024 Sep 6;13(17):2829. doi: 10.3390/foods13172829 (PMC11395680; doi:10.3390/foods13172829)
Supplement: Supplementary file 1 [file foods-13-02829-s001.zip › supplementary Table S2.pdf]

Table S2. Primer sequences used for vector construction

Gene name	Forward Primer	Reverse Primer
EjWRKY6-62SK	cgctctagaactagtggatccATGGACAAAGGATGGGGGC	gtcgacggtatcgataagcttTTAATTTCCCGGGAAGCTGC
EjWRKY6-PRI101	ttgatacatatgcccgtcgacATGGACAAAGGATGGGGGC	agagttgttgattcagaattcATTTCCCGGGAAGCTGCTAA
EjPSY1-0800	ttcctgcagcccgggggatccTTGGTGACTTCACATGACATTTATTT	tgtttttggcgtcttccatggGTTTGTTTCAACTTGAAAACACCAA
EjPSY2-0800	ttcctgcagcccgggggatccAAATTTATAGAATTATAAATCTCATAAAATACCAA	tgtttttggcgtcttccatggGAGCAATGTGCCTGAAACACAC
EjZISO-0800	ttcctgcagcccgggggatccTCCTGTTAGTTTTTAGGGTCAACAGT	tgtttttggcgtcttccatggTGAAAGTGGAGGAGGAGGAATCT
EjZDS2-0800	ttcctgcagcccgggggatccGAATTGCGTTGTCCAAACCG	tgtttttggcgtcttccatggGGATTCAAGCTTCTAAAAACCCAC
EjLCYB-0800	ttcctgcagcccgggggatccGTAGCACTTGAGCGTACTTATTTACACC	tgtttttggcgtcttccatggGAAATTCCAACACTTGTCCCGA
EjBCH2-0800	ttcctgcagcccgggggatccCTTTTATGAGAAATCTCTATTAAATTGGG	tgtttttggcgtcttccatggAGACCACAGCTTTCCGTCCC
EjZEP1-0800	ttcctgcagcccgggggatccTAAAAATAACCAATAGCCAGAAGATATTG	tgtttttggcgtcttccatggCTCTCTCCTCTCTCCCTCTCCAG
EjZEP3-0800	ttcctgcagcccgggggatccTCTGTTGGTTTTCTAGAGAGAGATTTG	tgtttttggcgtcttccatggTTGCCCGAATAGTGATACCTCC
